# Portal Vein Embolization as an Oncosurgical Strategy Prior to Major Hepatic Resection: Anatomic, Surgical, and Technical Considerations

**DOI:** 10.3389/fsurg.2016.00014

**Published:** 2016-03-11

**Authors:** Sonia T. Orcutt, Katsuhiro Kobayashi, Mark Sultenfuss, Brian S. Hailey, Anthony Sparks, Bighnesh Satpathy, Daniel A. Anaya

**Affiliations:** ^1^Section of Hepatobiliary Tumors, Department of Gastrointestinal Oncology, H. Lee Moffitt Cancer Center & Research Institute, Tampa, FL, USA; ^2^Diagnostic and Therapeutic Care Line, Section of Radiology, Michael E. DeBakey VA Medical Center, Baylor College of Medicine, Houston, TX, USA; ^3^Department of Radiology, Baylor College of Medicine, Houston, TX, USA

**Keywords:** portal vein embolization, hepatectomy, oncosurgical strategy, liver tumors, liver resection

## Abstract

Preoperative portal vein embolization (PVE) is used to extend the indications for major hepatic resection, and it has become the standard of care for selected patients with hepatic malignancies treated at major hepatobiliary centers. To date, various techniques with different embolic materials have been used with similar results in the degree of liver hypertrophy. Regardless of the specific strategy used, both surgeons and interventional radiologists must be familiar with each other’s techniques to be able to create the optimal plan for each individual patient. Knowledge of the segmental anatomy of the liver is paramount to fully understand the liver segments that need to be embolized and resected. Understanding the portal vein anatomy and the branching variations, along with the techniques used to transect the portal vein during hepatic resection, is important because these variables can affect the PVE procedure and the eventual surgical resection. Comprehension of the advantages and disadvantages of approaches to the portal venous system and the various embolic materials used for PVE is essential to best tailor the procedures for each patient and to avoid complications. Before PVE, meticulous assessment of the portal vein branching anatomy is performed with cross-sectional imaging, and embolization strategies are developed based on the patient’s anatomy. The PVE procedure consists of several technical steps, and knowledge of these technical tips, potential complications, and how to avoid the complications in each step is of great importance for safe and successful PVE and ultimately successful hepatectomy. Because PVE is used as an adjunct to planned hepatic resection, priority must always be placed on safety, without compromising the integrity of the future liver remnant, and close collaboration between interventional radiologists and hepatobiliary surgeons is essential to achieve successful outcomes.

## Introduction

With increasing understanding of liver vascular and biliary anatomy, advancements in surgical techniques, and use of intraoperative liver ultrasound, the safety of major hepatic resection has dramatically improved over the last few decades. Major hepatic resections are currently being performed at numerous hepatobiliary centers worldwide, with operative mortality rates of less than 2% (1, 2). Although surgical resection offers the best opportunity for long-term survival with the potential for cure in appropriately selected patients, extensive surgical resection can be associated with postoperative liver failure because of inadequate functional liver volume after surgery, especially when underlying liver disease is present and the extent of liver resection is significant.

Preoperative portal vein embolization (PVE) was developed to extend the indications for major hepatic resection, and it has become the standard of care for selected patients with hepatobiliary malignancies. By interrupting all of the portal vein flow to the diseased liver to be resected, this technique can induce atrophy of the diseased liver through removal of its primary blood supply, and through the release of specific hormones and growth factors (3–5), lead to hypertrophy of the non-diseased liver that will remain *in situ* after surgery [i.e., the future liver remnant (FLR)]. PVE also causes a shift in the function of the liver to the FLR and allows the patient to preoperatively adjust to changes in portal pressure to decrease the morbidity of the resection (6). The resultant increase in the functional volume of the FLR is geared to convert patients with unresectable disease into resection candidates, specifically by lowering the risk of postoperative liver insufficiency and failure (3, 5, 7, 8). In addition, the experience published at multiple centers worldwide has now established this technique to be safe and carry minimal morbidity (6, 9).

The indications for PVE depend upon the degree of underlying liver dysfunction and the extent of anticipated resection. Guidelines include for PVE to be performed when the FLR is anticipated to be ≤20% in normal livers, ≤30% in diseased livers or those in which chemotherapy-associated steatohepatitis (CASH) is suspected, and ≤40% in cirrhotic livers to reach those respective targets (6, 10). In addition, a degree of hypertrophy of at least 5% has been shown to predict outcome (6). Previous studies have determined that the FLR standardized to the patient’s size offers a good estimate of the functional liver volume; this is calculated with the FLR measured using computed tomography (CT) volumetry and calculating its contribution to the total liver volume as a proportion of the estimated total liver volume derived from the patient’s body surface area (BSA):

$$\begin{array}{l} \text{standardized FLR (sFLR)}\\ \text{=}\frac{\text{FLR }\left(\text{volume measured from CT}\right)}{\text{estimated total liver volume }\left(\text{ETLV}\right)} \end{array}$$

where ETLV = [−794.41 + 1267.28 × BSA in square meters].

This method limits inaccuracies related to the diseased liver (11, 12).

At present, there is no standardized technique used for PVE. Various techniques, with different embolic materials, have been used, without significant differences in the degree or rate of liver hypertrophy (9). Regardless of the techniques used, both surgeons and interventional radiologists must have essential knowledge universally applicable to PVE to communicate with each other and achieve successful outcomes for patients. First, a thorough knowledge of segmental liver anatomy and of the terminology used for hepatic resections is important to understand fully the segments that need to be embolized for anticipated hepatic resection. Second, knowledge of portal vein anatomy, especially that of the branching variations, is paramount to correctly select portal vein branches to embolize. Finally, knowledge of surgical techniques used for the anticipated hepatic resection is required because PVE performed without such knowledge can potentially render subsequent surgical procedures difficult, more risky, and/or occasionally result in the inability to accomplish a safe resection.

In this article, we review hepatic segmental anatomy and the terminology used for hepatic resections, portal vein anatomy, and branching variations, and the surgical techniques used for major hepatic resection. We discuss strategies for PVE based on portal vein anatomy and preprocedural planning with cross-sectional imaging. We also review and discuss approaches to the portal venous system, the embolic materials used, and the techniques and technical tips for safe and successful PVE. Finally, additional strategies or technical modifications that might be required during PVE are described.

## Hepatic Segmental Anatomy and Terminology Used for Hepatic Resections

The liver consists of eight functional segments, which have their own hepatic arterial and portal venous supply, biliary drainage, and hepatic venous drainage. As major hepatic resections require transection of the hepatic parenchyma along the boundaries of these segments (i.e., anatomic resection), understanding the hepatic segmental anatomy and the terminology used for segment-oriented hepatic resections is a prerequisite for PVE.

**Figure 1:**
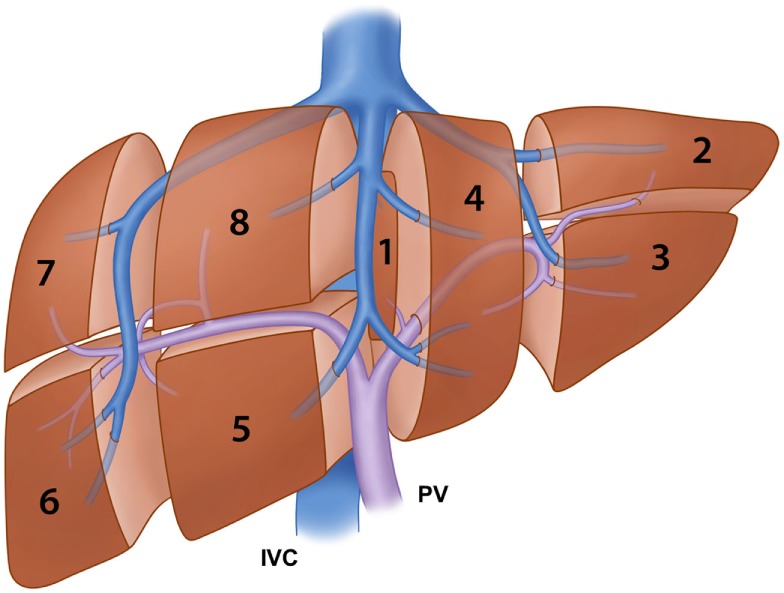
**Illustration of the segmental anatomy of the liver based on the third-order portal vein branching (Couinaud’s classification system) and the portal vein and hepatic vein branches**. IVC: inferior vena cava and PV: portal vein.

Several classification systems of liver segmentation have been proposed and used without a standardized terminology to describe the liver segments. Healey and Schroy (13) divided the liver into five segments based on second-order branching patterns of the hepatic arteries and bile ducts. Couinaud (14), whose classification system is likely the most commonly used worldwide, divided the liver into eight segments based on third-order portal vein branching. Goldsmith and Woodburne (15) also advocated division of the liver based on the portal vein branching; however, they described four segments based on second-order portal vein branching. Different terminology to describe the same functional unit of the liver, used by different researchers or anatomists, has resulted in much confusion among clinicians. In 2000, the terminology committee of the International Hepato-Pancreato-Biliary Association (IHPBA) proposed a standardized terminology for liver segmentation and hepatic resections during the World Congress of the IHPBA in Brisbane, Australia (16). In this proposal, the segmental liver anatomy was described according to the first-, second-, and third-order branching patterns of the hepatic arteries and bile ducts. The term “segment” (Sg) was restricted to indicating the third-order division, as in Couinaud segments 1–8 (Figure 1). The first-order division divides the liver into the left and right hemiliver or the left and right liver. The border, also called a watershed, of the first-order division is referred to as the midplane of the liver, which is defined by the plane between the inferior vena cava and the gallbladder fossa, also known as Cantlie’s line. The new term “section,” which is equivalent to “sector” in Couinaud’s classification system, was introduced to indicate the second-order division. The second-order division divides the liver into four sections. The right liver is divided into the right anterior section (Sg 5 and 8) and the right posterior section (Sg 6 and 7). The left liver is divided into the left lateral section (Sg 2 and 3) and the left medial section (Sg 4). The third-order division refers to the individual segments of the liver. The segments are referred to as segments 1–8. The segments are divided by intersegmental planes, which represent the borders of the segments.

**Figure 2:**
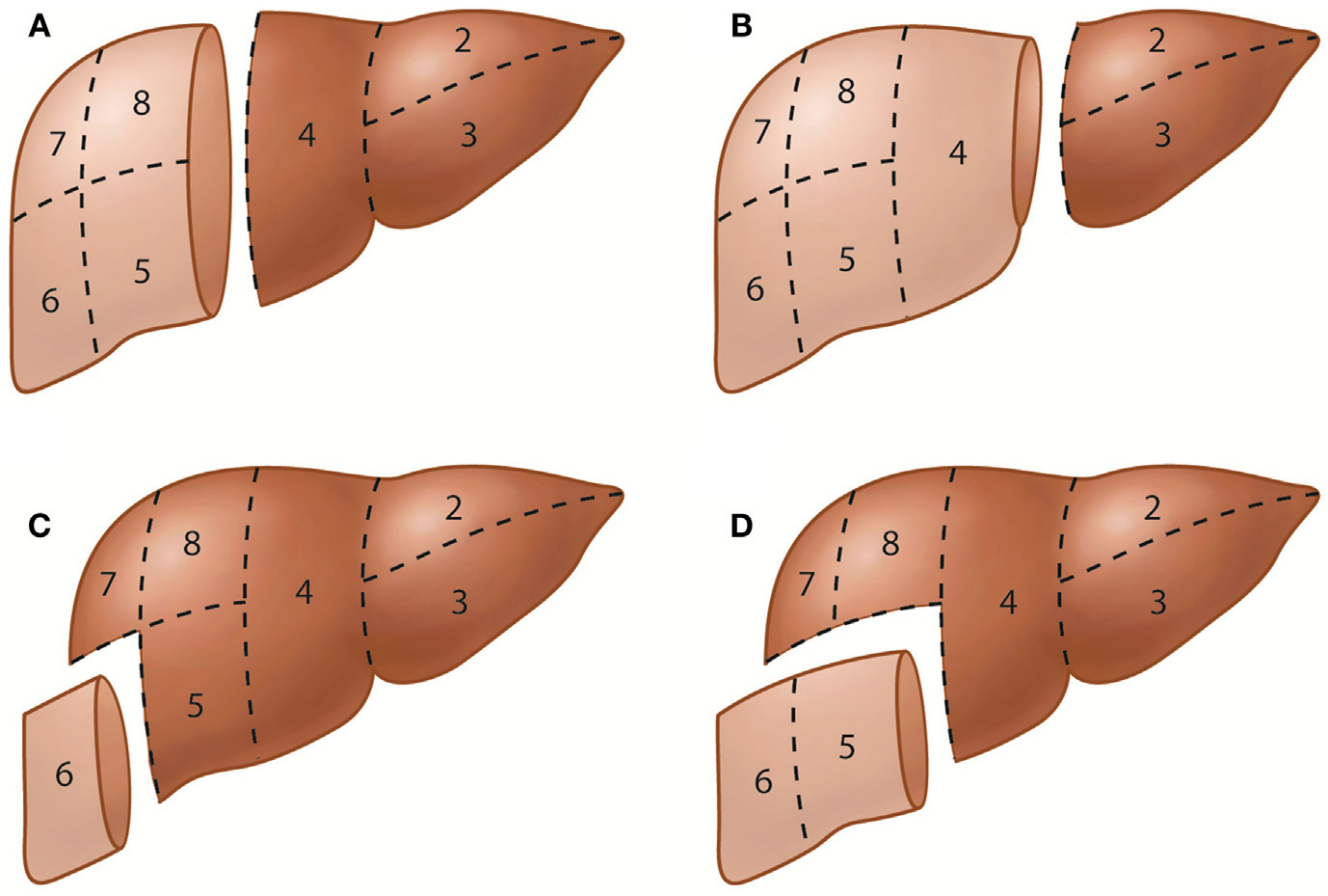
**Representative hepatic resections**. **(A)** Right hepatectomy or right hemihepatectomy (resection of segments 5–8) and left hepatectomy or left hemihepatectomy (resection of segments 2–4). **(B)** Right trisectionectomy or extended right hepatectomy (resection of segments 4–8) and left lateral sectionectomy or bisegmentectomy 2 and 3 (resection of segments 2 and 3). **(C)** Segmentectomy 6 (resection of segment 6). **(D)** Bisegmentectomy 5 and 6 (resection of segments 5 and 6).

The terminology of hepatic resections is based directly on the anatomic terminology used in this proposal (Figure 2). Resection at the first-order division is called right or left hepatectomy or hemihepatectomy. Resection of a single section is referred to as sectionectomy (i.e., left lateral sectionectomy). Extended resections of three sections are called trisectionectomies (or extended hepatectomies/hemihepatectomies). Resection of a single segment is referred to as segmentectomy (i.e., segmentectomy 6), and resection of any two contiguous segments is referred to as a bisegmentectomy (i.e., bisegmentectomy 5 and 6) (16). When segment 1 is resected as part of the procedure, it should be stated as in the following examples: “left hepatectomy with resection of segment 1” or “left hepatectomy extended to segment 1.” The Brisbane 2000 terminology of liver anatomy and resections has been increasingly used in the literature worldwide (17); adherence to this terminology is encouraged to avoid confusion when referring to liver segments or hepatic resections.

## Normal Portal Anatomy and Portal Branching Variations

**Figure 3:**
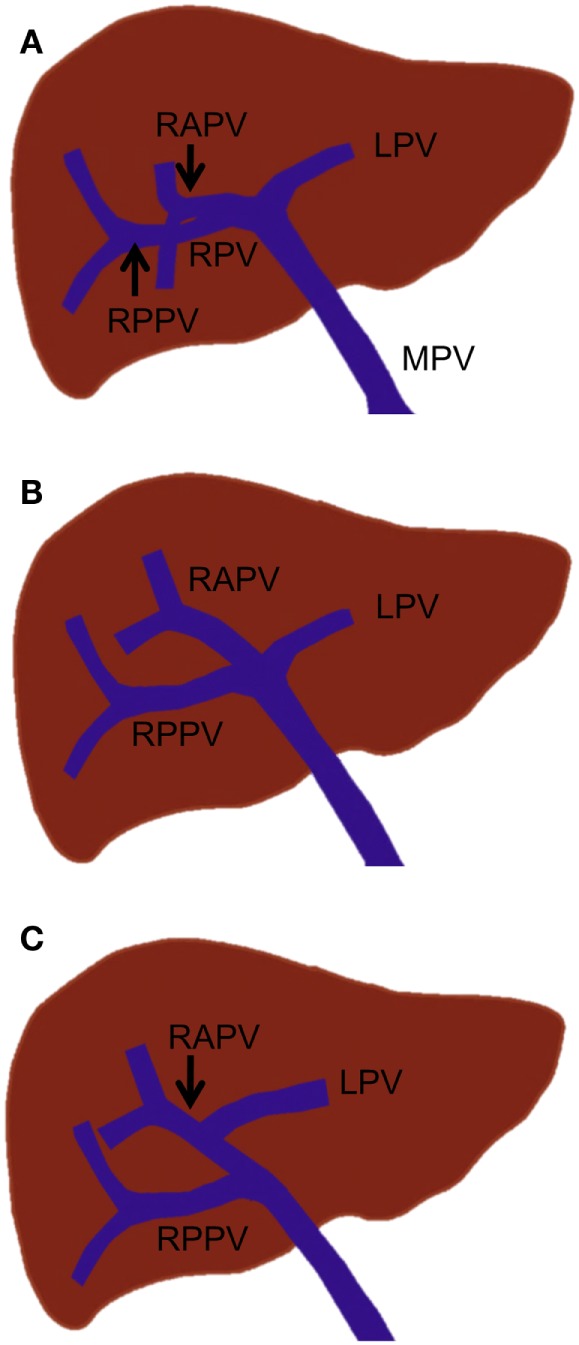
**Standard portal vein branching and main portal vein branching variations**. **(A)** Standard portal vein branching. **(B)** Trifurcation of the main portal vein. **(C)** The right posterior portal vein arising as the first branch of the main portal vein. MPV, main portal vein; LPV, left portal vein; RPV, right portal vein; RAPV, right anterior portal vein; and RPPV, right posterior portal vein.

In standard portal vein anatomy, the main portal vein ascends through the hepatoduodenal ligament and typically divides at the hepatic hilum into the left and right portal veins. The left portal vein courses medially to the umbilical fissure, forming the horizontal part (*pars transversa*), and then turns anteriorly to form the umbilical part (*pars umbilicalis*). The left portal vein gives off the branches supplying Sg 2 (Sg 2 branch) and Sg 3 and 4. The right portal vein gives rise to the right anterior and posterior portal veins, which supply the anterior and posterior sections, respectively. The right anterior portal vein further divides into the Sg 5 and 8 branches, while the right posterior portal vein divides into the Sg 6 and 7 branches (Figure 3A). This standard portal vein anatomy is observed in approximately 65–80% of the population, and any deviation from this anatomy is considered to be a branching variation (18–20).

The most common main portal vein branching variations are trifurcation of the main portal vein (Figure 3B) and the right posterior portal vein arising as the first branch of the main portal vein (Figure 3C). The reported incidence of these branching variations has ranged from 8 to 23% (18, 19, 21, 22). Quadrification, in which the main portal vein divides into the left portal vein, right anterior portal vein, and Sg 6 and 7 branches, can occur, but it is quite uncommon. Other rare main portal vein variations have been described, including duplication or absence of the right portal vein, which is associated with a hypoplastic right liver and absence of the horizontal part of the left portal vein, in which a single portal vein enters the right liver and courses to the left, giving only segmental branches along its course (23).

**Figure 4:**
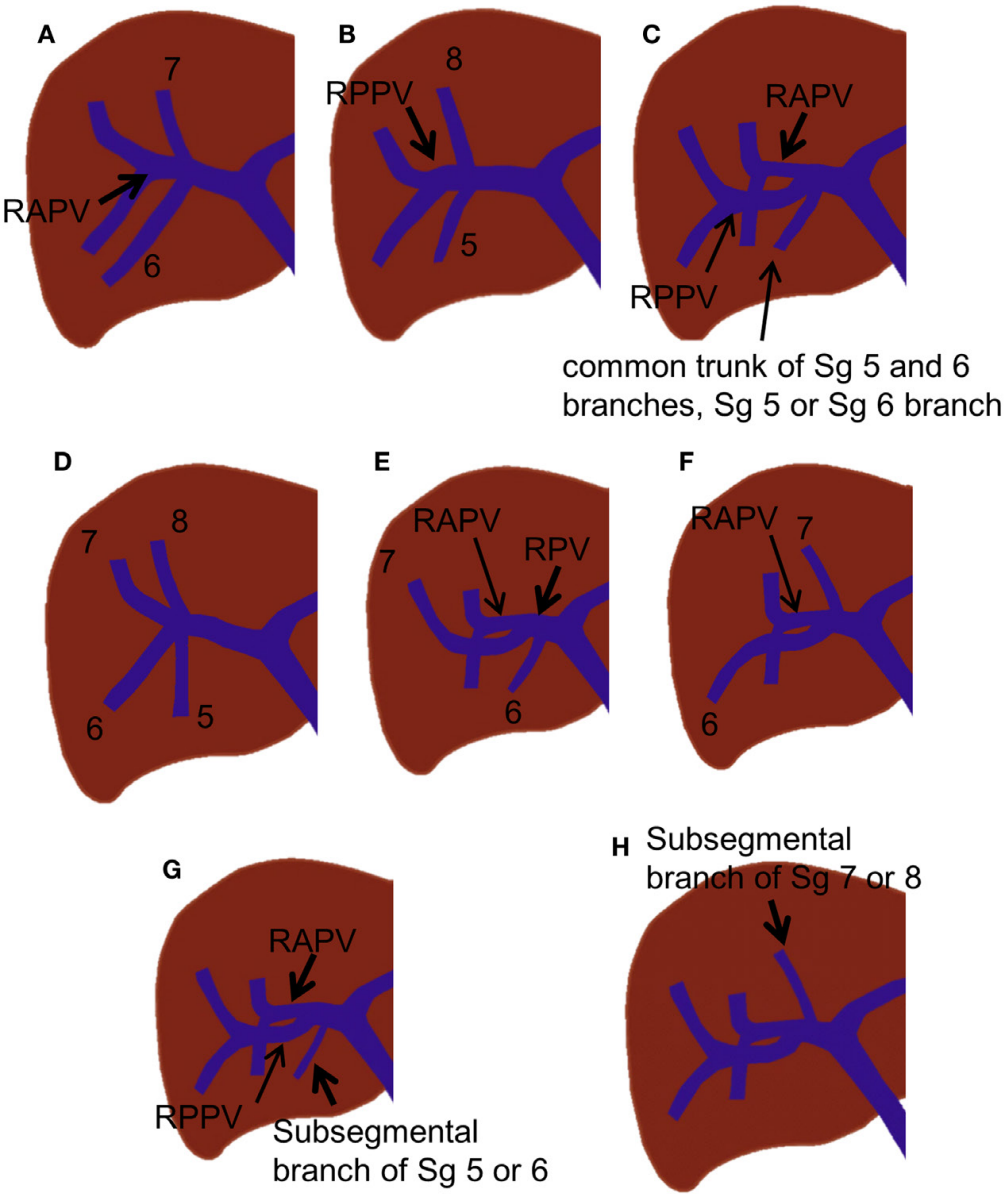
**Right portal vein branching variations**. **(A–C)** Trifurcation of the right portal vein. **(D)** Quadrification of the right portal vein. **(E,F)** Proximal origin of the segmental branch from the right portal vein. **(G,H)** Proximal origin of the subsegmental branch from the right portal vein.

Various branching variations of the right portal vein have been reported, with incidences ranging from 17 to 35% (19, 24, 25). The most common variations are trifurcation of the right portal vein and proximal origin of a segmental branch from the right portal vein. The right portal vein has been reported to trifurcate in several ways; however, the most common type is trifurcation into the right anterior portal vein and Sg 6 and 7 branches without a common trunk of the right posterior portal vein (Figure 4A) (18, 19, 25). Other types of trifurcation may be encountered, such as division into the right posterior portal vein and Sg 5 and 8 branches (Figure 4B); right anterior and posterior portal veins and a separate common trunk of the Sg 5 and 6 branches; or right anterior or posterior portal veins and separate Sg 5, 6, or 8 branches (Figure 4C); however, these variations are less frequent (19, 25). Rarely, the right portal vein divides into four separate segmental branches (Sg 5–8) without a common trunk of the right anterior and posterior portal veins (Figure 4D). A segmental or subsegmental branch can often originate from the right portal vein, proximal to the bifurcation of the right anterior and posterior portal veins. Of the segmental branches originating from the right portal vein, the Sg 6 and 7 branches are the most commonly observed (Figures 4E,F). The subsegmental branches supplying segments 5–8 can also originate from the right portal vein (Figures 4G,H).

**Figure 5:**
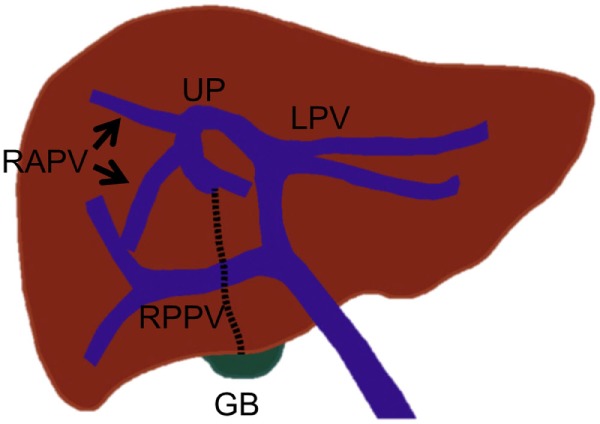
**Drawing illustrates the right anterior portal vein that arises from the left portal vein in an anomaly with the right-sided ligamentum teres that is juxtaposed to the gallbladder**. In this anomaly, the umbilical portion of the left portal vein is also deviated to the right. UP, umbilical portion; LT, ligamentum teres (dotted line); and GB, gall bladder.

**Figure 6:**
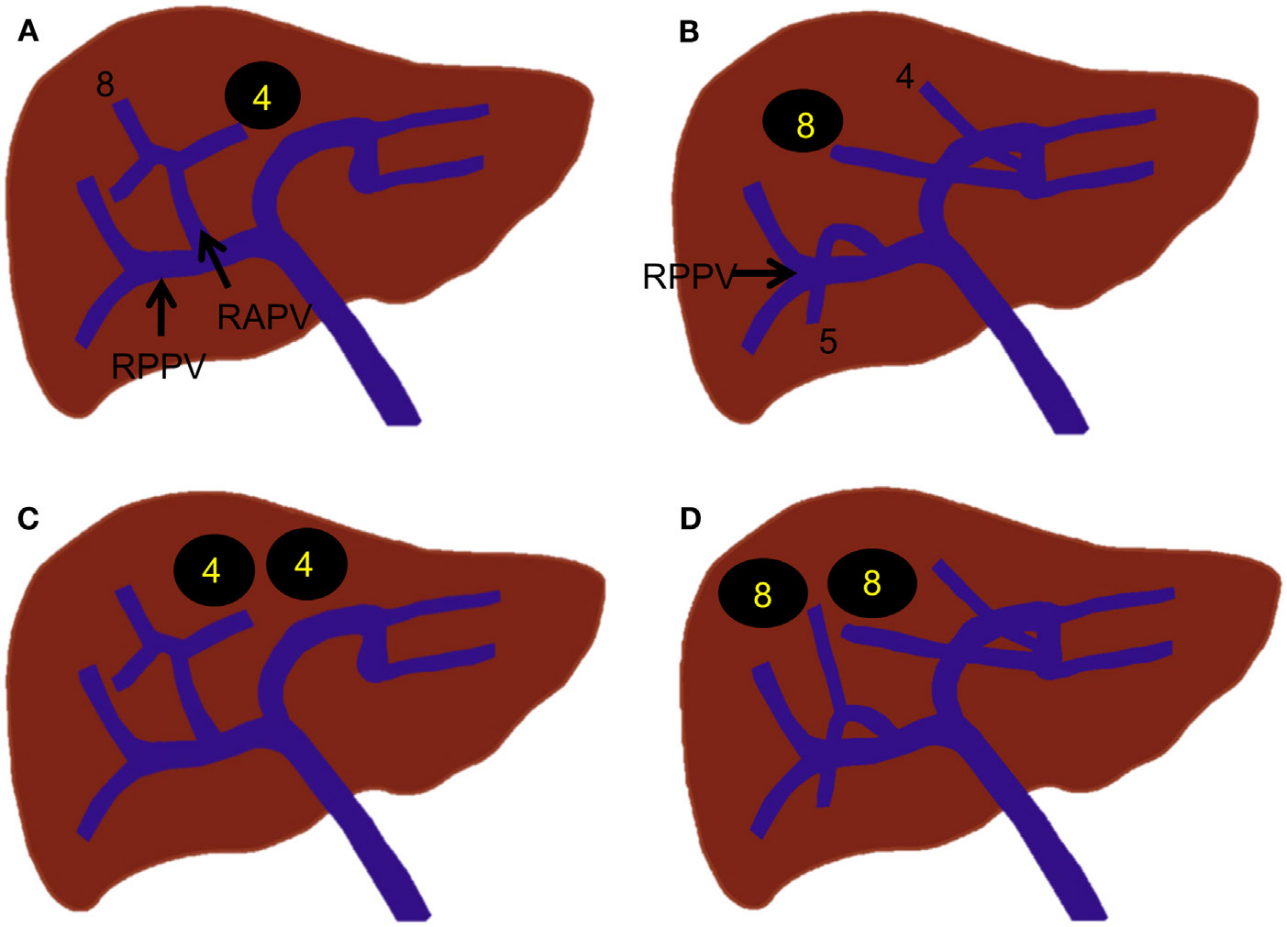
**Segmental or subsegmental portal vein branches that cross the midplane of the liver**. **(A)** A Sg 4 branch that arises from the right anterior portal vein. **(B)** A Sg 8 branch that arises from the left portal vein. **(C)** A subsegmental branch that arises from the right anterior portal vein but supplies Sg 4. **(D)** A subsegmental branch that arises from the left portal vein but supplies Sg 8.

The portal vein branches crossing the midplane of the liver (which separates the right and left liver) are rare, but they are very important to recognize prior to PVE. If these branches are unrecognized, the left portal vein branches that supply the diseased right liver can be inadvertently left unembolized, or conversely, the right portal vein branches that supply the non-diseased left liver can be inadvertently embolized during right PVE (embolization of Sg 5–8). In a portal vein anomaly, in which the ligamentum teres is deviated to the right and is juxtaposed to the gallbladder, the right anterior portal vein arises from the rightward-deviated umbilical portion of the left portal vein (Figure 5) (26). In this anomaly, the main portal vein first gives off the right posterior portal vein, courses cranially without forming the horizontal part, and terminates at the rightward-deviated umbilical portion. This anomaly has been reported to be encountered in less than 1% of patients. Some authors have referred to this anomaly as “fusion of hepatic planes,” as they hypothesize that this anomaly is caused by fusion of the midplane of the liver with the left intersectional plane due to incomplete development of the central part of the liver (27, 28). A segmental or subsegmental portal vein branch can also cross the midplane of the liver (Figure 6). The Sg 8 branch can arise from the left portal venous system, or the Sg 4 branch can arise from the right portal venous system. Very rarely, the Sg 6 or 7 branch arises from the left portal venous system (25). The diameters of these variant branches have been reported to range from 0.5 to 2.7 mm, and these branches are encountered in less than 4% of patients (22).

## Surgical Techniques Used for Major Hepatic Resection

For those patients receiving preoperative PVE, the most common resection is either a right hepatectomy (resection of Sg 5–8) or a right trisectionectomy (resection of Sg 4–8). Patients undergoing hepatic resections other than these two resections do not usually require preoperative PVE because they will normally have a sufficiently functional FLR, which does not necessitate PVE. Left trisectionectomy (Sg 2–5 and 8) only rarely requires preoperative PVE, most commonly when performed on patients with liver disease. Therefore, the surgical techniques used for right hepatectomy and right trisectionectomy will be discussed herein. Because a review of the technical details of hepatic resections is beyond the scope of this article, only the essential technical steps, especially those relevant to PVE, are highlighted.

**Figure 7:**
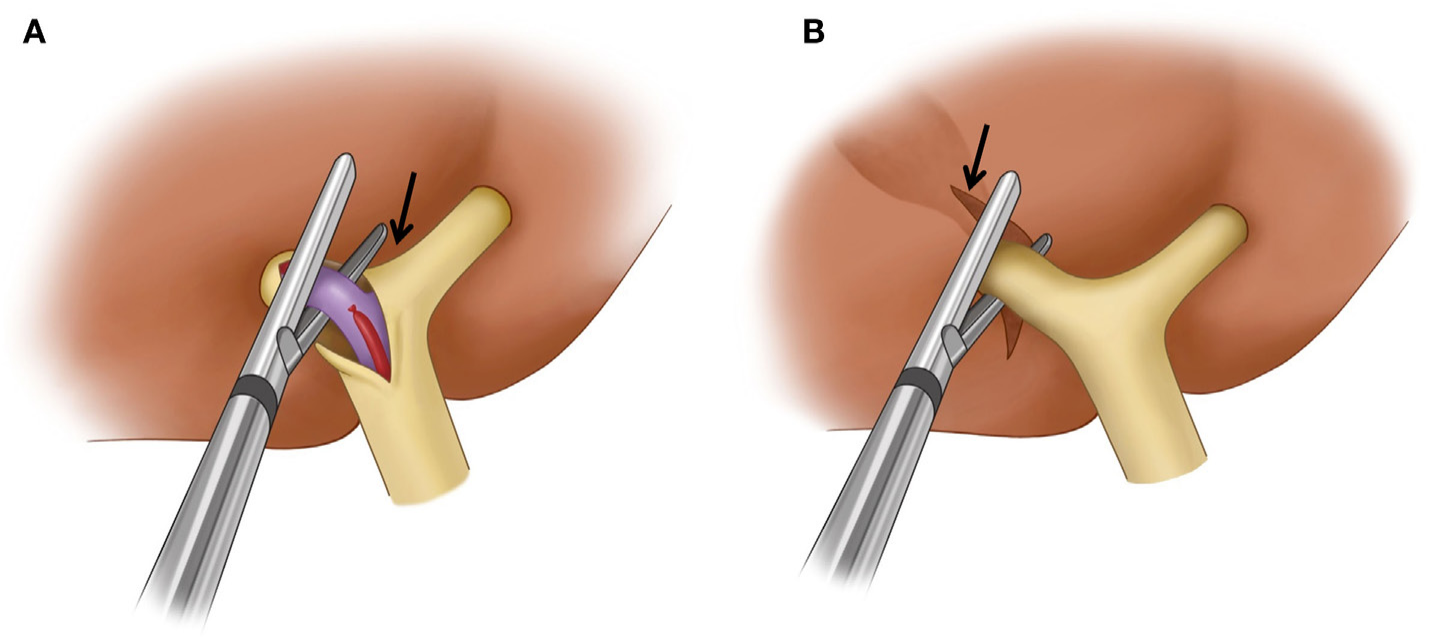
**Surgical techniques used for the control of inflow to the liver in a right hepatectomy**. **(A)** Extrahepatic dissection and ligation. The hilar plate (arrow) is dissected and the right hepatic artery and right portal vein are ligated and dissected individually. A vascular stapler is used to divide the right portal vein. **(B)** Intrahepatic pedicle ligation. Arrow indicates the site of a hepatotomy made at the inferior surface of the liver to expose the intrahepatic main right portal pedicle. A vascular stapler which is inserted into the liver parenchyma divides the main right portal pedicle.

Although there are many different techniques and sequences used for major hepatic resections, any anatomic hepatic resection is generally performed according to three principles: (1) control of inflow (portal vein and hepatic artery – and biliary ducts) to the liver, (2) control of outflow (hepatic vein) from the liver, and (3) parenchymal transection (29). Control of inflow to the liver can be achieved extrahepatically at the hepatic hilum (extrahepatic dissection and ligation). The thick connective tissue that wraps around the portal vein, hepatic artery, and bile duct at the hepatic hilum (hilar plate) is dissected first. The right hepatic artery and right portal vein are then isolated and ligated individually (Figure 7A). This is the classic approach to inflow vessel control. Alternatively, inflow to the liver can be controlled intrahepatically through a hepatotomy made at the inferior surface of the liver to access the portal pedicle within the liver (intrahepatic pedicle ligation/intrahepatic Glissonian approach). The portal pedicle or Glissonian pedicle is a vascular pedicle that contains the portal triad. This pedicle is wrapped with thick connective tissue that is contiguous with the hilar plate. Because the portal pedicle ramificates within the liver to supply the segment or section into which the pedicle enters, any segment or section can be safely resected by ligation of its corresponding pedicle (30). In a right hepatectomy, the right main portal pedicle within the liver parenchyma is isolated and ligated (Figure 7B). A vascular stapler is often used to divide the portal pedicle. Intrahepatic pedicle ligation offers some advantages over the extrahepatic dissection and ligation because it eliminates the need for hilar dissection and reduces the risk of injury to the vessels or biliary drainage of the contralateral liver. When performing PVE, care must be taken to avoid placing embolic material, such as coils, in locations that may limit the surgeon’s ability to divide the portal vein at the desired location. For instance, if the surgeon routinely uses an extrahepatic dissection and ligation technique, it is helpful to leave the proximal 1-cm portion of the right portal vein free of embolic material so that the right portal vein can be easily ligated and divided. Surgeons who use an intrahepatic pedicle ligation technique might prefer to ligate the right anterior and posterior portal pedicles individually. In such cases, embolic materials should not be left within the proximal right anterior or posterior portal veins. Conversely, the surgeon needs to be knowledgeable and cognizant about the location of the coils and embolic material, as if the PVE procedure did not allow to place the embolic material in the specific desired location, as described, the surgeon will need to adjust the surgical technique to accomplish the desired resection in a safely manner. Therefore, knowledge of the inflow control technique the surgeon plans to use during the procedure and understanding details of the PVE procedure performed by the interventional radiologist are both critical pieces of information when planning PVE and subsequent surgery; and discussion between the two parties regarding the most proximal site of the portal veins into which embolic materials can be placed is mandatory prior to PVE. Control of outflow from the liver is performed by isolating and dividing the right hepatic vein and the small hepatic veins that drain directly into the inferior vena cava. Once inflow to and outflow from the liver are controlled, the liver is transected along the line demarcated by the devascularization of the right hemiliver resulting from division of the right hepatic artery and right portal vein (parenchymal transection). Some surgeons may control inflow during parenchymal transection by dividing portal pedicles with a vascular stapler as they are encountered during transection. In this technique, the hepatotomy at the inferior surface of the liver to isolate the right main portal pedicle is not necessary.

In right trisectionectomy, the initial steps are the same as with right hepatectomy, up through control of inflow to and outflow from the liver. The next step involves selective ligation of the portal pedicles supplying Sg 4. The liver parenchyma is then transected immediately to the right of the falciform ligament. The middle hepatic vein is generally left intact until it is encountered in the upper part of the dissection, at which point it is either suture- or staple-ligated.

## Embolization Strategies Based on Portal Branching Anatomy

**Figure 8:**
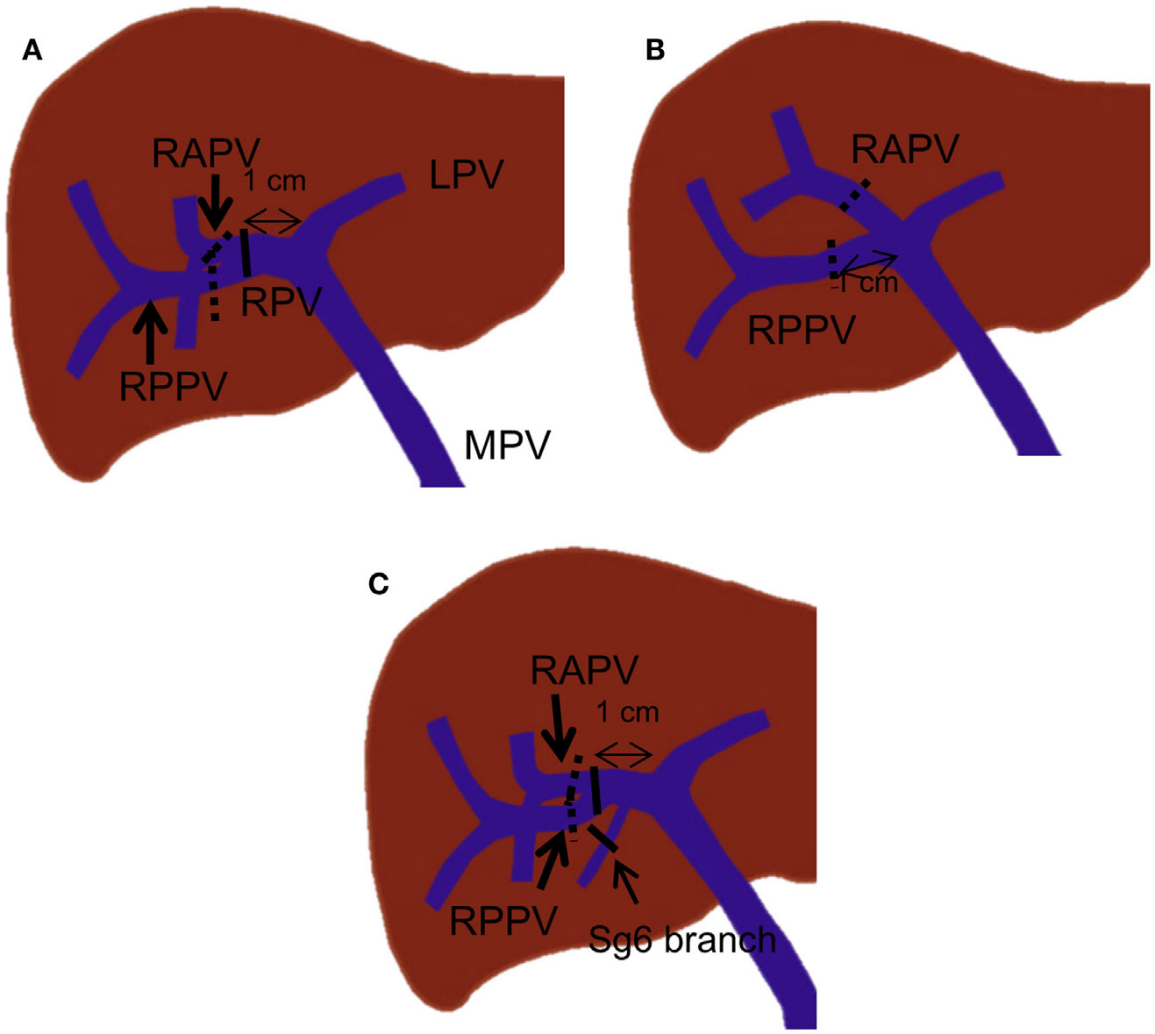
**Right PVE strategies based on portal vein branching anatomy**. In each drawing, black lines indicate the most proximal site or sites where embolic materials can be placed when the length of the right portal vein is more than 1 cm. The dotted black lines indicate the most proximal site or sites where embolic materials can be placed when no right portal vein exists or the length of the right portal vein is less than 1 cm. **(A)** A case with standard portal vein anatomy. **(B)** A case with trifurcation of the main portal vein. **(C)** A case with a Sg 6 branch arising from the proximal 1-cm portion of the right portal vein.

In general, strategies for PVE are devised based on the type of surgery planned, the surgeon’s preference for the technique used for vascular inflow control (extrahepatic vs. intrahepatic), and the portal branching anatomy. If right hepatectomy is planned, many authors have recommended that a proximal right portal vein at least 1 cm in length remain patent to facilitate hepatic resection (31, 32). This recommendation also reduces the risk of propagation of right portal vein thrombus to the left portal vein. In standard portal vein anatomy, in which the main portal vein divides into the right and left portal veins, the length of the right portal vein ranges from 0.5 to 2.3 cm (33, 34). If the length of the right portal vein is greater than 1 cm, the most proximal site where the embolic material should be placed is within the distal right portal vein (Figure 8A). If the length of the right portal vein is less than 1 cm, the right anterior and posterior branches must be embolized individually (Figure 8A). Individual embolization of the right anterior and posterior branches might also be necessary if the surgeon prefers the entire right portal vein to be patent or if he or she uses the intrahepatic pedicle ligation technique, in which the distal right portal vein is normally ligated.

For patients with portal vein branching variations, different strategies are required. For instance, in patients with main portal vein trifurcation in which no right portal vein exists, the right anterior and posterior portal veins must be embolized individually, with at least a 1-cm proximal portion remaining patent (Figure 8B). In patients with a segmental branch arising from the right portal vein, this branch usually needs to be embolized separately, especially when the branch arises from the proximal 1-cm portion of the right portal vein (Figure 8C).

## Approaches to the Portal Venous System

Access to the portal venous system can be accomplished by any of three approaches: transhepatic contralateral, transhepatic ipsilateral, and transileocolic approaches. Each approach has its own advantages and disadvantages (Table 1). The choice of the approach can depend on the type of surgery planned, the embolic material used, the operator’s preference, and the level of the operator’s experience with one approach over the others.

**Table 1:** **Characteristics of three approaches used for PVE**.

Approach to the portal system	Technique	Advantages	Disadvantages
Contralateral approach	Transhepatic portal vein cannulation within the non-diseased liver (FLR)	Easy catheter manipulation within the diseased liver	Potential damage to the FLR during portal access or catheter manipulation
		Theoretically no risk of dislodging embolic materials during final portography	Difficult cannulation of Sg 4 portal branches for embolization when a right trisectionectomy is anticipated
Ipsilateral approach	Transhepatic portal vein cannulation within the diseased liver to be resected	Theoretically no risk of damage to the FLR related to portal access or catheter manipulation	Technically challenging catheter manipulation within the portal branches in the diseased liver
			Possible migration of embolic materials into the FLR during final portography
Transileocolic approach	Direct cannulation of the ileocolic vein during laparotomy	Accurate assessment of peritoneal seeding and disease extension to the FLR	Most invasive Requires general anesthesia Exposed to post-laparotomy complications
		Theoretically no risk of damaging the FLR, hemoperitoneum, or subcapsular hematoma

*FLR, future liver remnant*.

The transhepatic contralateral approach involves puncturing the portal vein branch within the FLR under ultrasound guidance. The catheter is then advanced into the portal venous system within the diseased liver for embolization. When right PVE (embolization of Sg 5–8) is performed, this approach allows for technically smooth cannulation of the right portal vein from the left liver. If *N*-butyl cyanoacrylate (NBCA) is used for embolization, this approach is desirable because NBCA can be delivered to the distal portal branches in an antegrade manner, without the risk of catheter entrapment within the portal venous system. A final portogram can be obtained with a minimal risk of dislodging the embolic material. In addition, there is no theoretical risk of puncturing the tumor tissue, which can result in bleeding or tumor seeding. One major disadvantage of this approach is the potential risk of damaging the FLR parenchyma and the left portal vein because of direct puncture of and instrumentation within the portal vein in the FLR. Should these events occur, the anticipated hepatic resection could become difficult or even impossible. When this approach is used, great care must be exercised to limit the number of punctures of the portal vein branch, and gentle catheter manipulation within the FLR is mandatory. Tract embolization is not usually performed to avoid non-targeted embolization.

The transhepatic ipsilateral approach involves puncturing the portal vein branch within the diseased liver to be resected under ultrasound or fluoroscopic guidance. The main advantage of this approach is the much lower risk of damaging the FLR compared to the contralateral approach because the FLR is not punctured and instrumented. With this approach, the complications that can arise from the PVE procedure rarely result in abandonment of the planned hepatic resection. An additional advantage of the ipsilateral approach is technically easier catheterization of Sg 4 compared to the contralateral approach, when Sg 4 needs to be embolized. The disadvantages of the ipsilateral approach include technically difficult cannulation of the right portal vein branches because of acute angulations between the branches and the risk of migration of embolic material during the final portography or catheter removal. To overcome the difficult cannulation of the right portal vein branches, a reverse curve catheter or an occlusion balloon catheter with multiple lumens is usually needed. Great care must be exercised not to dislodge the embolic material during or after embolization of the portal vein branches.

The transileocolic approach involves a laparotomy focused on the right lower quadrant and direct cannulation of the ileocolic vein. The catheter is advanced into the portal vein branches through the main portal vein for embolization. This approach was the original PVE method used by surgeons; however, it has not gained widespread use with the development of percutaneous transhepatic approaches. Although the transileocolic approach has several disadvantages that inherently arise from a laparotomy, it has some advantages over the transhepatic approaches. This approach enables the operator to detect peritoneal seeding and additional staging procedures, which may result in advanced disease beyond a stage amenable to surgical treatment, and hence making the PVE itself and the planned surgery unnecessary (35). In cases with multiple or large liver tumors, the transileocolic approach might be ideal to avoid puncturing the tumors while obtaining portal access.

## Embolic Materials Used for PVE

Knowledge of the embolic materials available for PVE is a prerequisite for secure, durable embolization without complications. A broad spectrum of embolic materials have been used, including NBCA, absolute ethanol, microparticles, gelatin sponge or Gelfoam^®^, fibrin glue, or combinations of these materials with coils or vascular plugs. Their advantages and disadvantages when used for PVE and other characteristics are shown in Table 2. In animal models, several researchers have demonstrated the significant differences in the degrees of hepatocyte proliferation among various embolic materials (36, 37). However, neither clinical studies have revealed significant differences in the efficacy of one embolic material over another in inducing liver hypertrophy nor have differences in complication rates been demonstrated among the various embolic materials. Currently, there is no consensus regarding the best embolic material for PVE (38). Rather, the choice of embolic material is based on the operator’s preference and experience with embolic material, the catheter available for the delivery of the embolic material, or the approach to the portal venous system used for PVE.

**Table 2:** **Characteristics of embolic materials used for PVE**.

Embolic material	Advantages	Disadvantages	Other characteristics
NBCA	Durable portal occlusion that lasts for more than 4 weeks	Peribiliary fibrosis and casting of the portal vein that could increase surgical difficulty	Mixed with ethiodized oil at a ratio of 1:1–1:3
	Can be associated with a shorter procedure time than with other materials	Requires a high level of experience	
		Difficult to use in patients with reduced hepatopetal flow	
Microparticles (PVA or microspheres)	Durable portal occlusion if used with coils	Usually requires many bottles if small-sized particles are used alone	Frequently used with coils to occlude proximal portal veins
	Easy to administer		
	Minimal periportal reaction		
Absolute ethanol	Durable portal occlusion	Requires an occlusion balloon catheter to administer	Good hypertrophy reported in patients with chronic liver disease
		Causes hepatocyte necrosis, resulting in transaminase and bilirubin elevation	
		Less patient tolerability because of associated pain	
Gelatin sponge/Gelfoam^®^	Easy to administer	Associated with a high rate of portal recanalization if used alone	Can be mixed with ethiodized oil or thrombin
	Inexpensive		
	Minimal periportal reaction		
Fibrin glue	Reported favorable rates of hypertrophy in Eastern countries	Requires a special balloon catheter with separate lumen to administer	Mixture with ethiodized oil necessary for radiopacity
		Portal recanalization can occur within 4 weeks if used alone	

*NBCA, *N*-butyl cyanoacrylate; PVA, polyvinyl alcohol*.

*N*-butyl cyanoacrylate is a radiolucent liquid agent that polymerizes within seconds in ionic solutions, such as blood. NBCA is mixed with ethiodized oil, normally at a ratio of 1:1–1:3, for slower polymerization and radiopacity. By changing the NBCA-to-ethiodized oil ratio, the time to polymerization can be adjusted, depending on the portal flow velocity and the order of the portal veins targeted for embolization. To embolize distal portal vein branches, a higher ratio mixture is used as opposed to a lower ratio mixture to embolize the proximal portal vein branches. The delivery catheter must be frequently flushed with a non-ionic solution (such as 5% dextrose) to prevent occlusion of the catheter lumen. Several clinical studies have shown that embolization with NBCA results in fast, reliable, and long-term portal occlusion and leads to good hypertrophy of the FLR (39, 40); however, use of NBCA requires considerable technical expertise and can make subsequent hepatic surgery challenging due to the significant periportal inflammation it causes. Absolute ethanol is a strong sclerosant and causes protein denaturation and coagulation. Embolization with absolute ethanol has been shown to produce reliable hypertrophy of the FLR, even in patients with chronic liver disease (41, 42). However, absolute ethanol can cause significant periportal necrosis and fibrosis and greater alteration in measured liver function tests can result when compared to PVE with other embolic materials. Potential systemic effects with absolute ethanol, such as intoxication or abdominal pain, might be associated with poor patient tolerance. Microparticles, such as polyvinyl alcohol (PVA) or tris-acryl microspheres, can accurately target the vessels to be occluded within the vascular network in the tumor or organ. Microparticles are available in a variety of sizes, mostly ranging from 100 to 1200 μm in diameter. They are generally easy to use, cause minimal periportal reaction, and generate durable portal occlusion when used in combination with coils (43). The theoretical benefit of using this embolic material is a smaller chance of developing a collateral blood supply within the embolized segments because of distal portal branch embolization. Gelatin sponge or Gelfoam^®^ is a water-insoluble temporary embolic material that can be used as a form of injectable pledgets or slurry. This material, which is used as a hemostatic device, is widely available and inexpensive. If used alone, recanalization can occur in 2 weeks after PVE and inadequate hypertrophy can result from embolization with gelatin sponge (44, 45). However, when combined with other embolic materials, such as polidocanol or ethiodized oil, favorable hypertrophy rates comparable to those observed with other embolic materials have been reported (46, 47). Fibrin glue, a mixture of fibrinogen and thrombin, was designed to mimic the final steps of the coagulation cascade and forms stable, physiological fibrin clots that assist hemostasis. It is used with ethiodized oil to confer radiopacity. Hypertrophy rates comparable to the rates measured with other embolic materials have been reported (31, 48). However, a specialized balloon catheter, with separate lumens for the delivery of both fibrinogen and thrombin, is necessary to administer fibrin glue, and this catheter is not available worldwide. Finally, foam sclerotherapy using polidocanol has recently been reported to be safe and effective for PVE (49). In the study, polidocanol foam was administered into the targeted vessels through the occlusion balloon catheter. Because of polidocanol’s anesthetic effect, PVE with polidocanol foam was well tolerated without pain and the sufficient degrees of liver hypertrophy that are comparable to those reported with other embolic materials were observed.

## Preprocedural Planning with Cross-Sectional Imaging

As applicable to all complex interventional procedures, preprocedural planning with cross-sectional imaging is a prerequisite for successful PVE. Multiphasic multidetector-row CT or dynamic contrast-enhanced magnetic resonance imaging is usually obtained before PVE to evaluate the extent of the liver disease and to measure the volume of the FLR. The portal and hepatic vein anatomy is assessed, and the segments to be resected are confirmed. Portal vein variations relevant to anticipated PVE are identified. Attention must be paid to identify any portal vein branches that cross the midplane of the liver or intersegmental boundaries. As discussed in the previous section, the portal branches that supply the liver segments to be resected but that arise from the FLR can be easily left unembolized, or conversely, the branches that supply the FLR but that arise from the liver segments to be resected can be inadvertently embolized. Once all of the portal branches that supply the liver segments to be resected are confirmed, the most proximal sites of the portal vein branches in which embolic material can be placed are identified. These sites can vary depending on the portal vein branching anatomy, the length of the right portal vein, and the surgical techniques used for control of inflow to the liver, as discussed in the previous section. The diameters of the portal branches to be embolized are measured to estimate the size of the occlusion balloon used or that of embolization coils to be placed. Finally, the distal branches of the portal venous system are assessed for safe portal access. If an ipsilateral approach is planned, the anticipated portal access tract should not transgress the tumor. If no ideal distal portal vein branch can be identified for access due to anatomical distortion of the portal vein branches from a large tumor or the presence of multiple tumors, then a contralateral approach might need to be considered.

## Techniques and Tips for Percutaneous PVE

Regardless of the different techniques and embolic materials used, PVE generally consists of five basic technical steps: (1) gaining access to the portal venous system, (2) flush portography and portal pressure measurement, (3) catheterization of the portal vein branches within the liver segments to be resected and delivery of embolic material to the branches, (4) final flush portography and portal pressure measurement, and (5) removal of the devices used, with or without tract embolization. Knowledge of technical tips, potential complications, and how to avoid the complications in each step of PVE is of great importance to perform the procedure safely and successfully.

**Figure 9:**
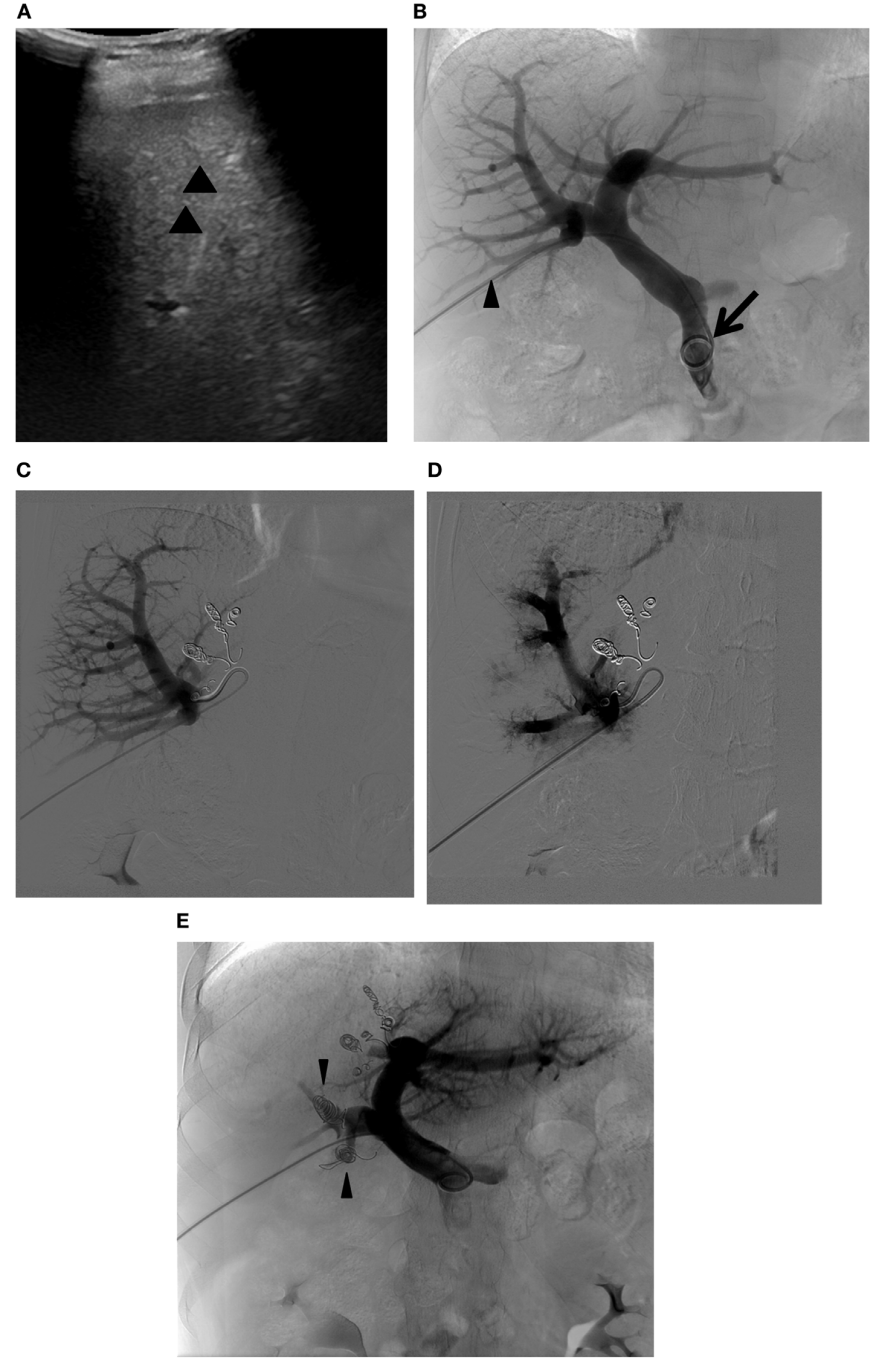
**Right PVE extended to segment 4**. **(A)** Intraprocedural ultrasound image shows a 22-gauge Chiba needle (arrowheads) placed within the branch of the right portal vein. A metal introducer set was subsequently introduced into the right portal system over a 0.018 guidewire (not shown). **(B)** Flush portogram shows a 5-Fr pigtail catheter (arrow) introduced through a 6-Fr sheath (arrowhead) and placed within the main portal vein. No variant main portal vein branching is demonstrated. **(C)** Selective right portogram, using a 5-Fr reverse curved catheter, shows right portal vein branches. Note the metallic coils placed within the Sg 4 branches. **(D)** Selective right portogram following delivery of PVA particles into the right portal vein branches shows embolized subsegmental branches within the right liver. **(E)** Final portogram shows successful embolization of the right portal vein and Sg 4 branches, with preserved blood flow to the FLR (Sg 2 and 3). Note the metallic coils placed within the proximal right anterior and posterior branches (arrowheads).

The first step in PVE is to gain access to the portal venous system. Ultrasound is used to identify a suitable peripheral portal vein branch for safe access. It is the authors’ preference that the peripheral portal vein branch be punctured with a 22-gauge Chiba needle (Cook Medical, Bloomington, IN, USA) under ultrasound guidance. A metal introducer set (SKATER™ Introducer System, Angiotech, Gainesville, FL, USA) is then advanced into the portal vein branch over a 0.018″ guidewire, followed by placement of a 5- or 6-Fr vascular sheath to establish portal access. The vascular sheath must be sufficiently long to reach the portal vein branch. Otherwise, access to the portal venous system will be lost when the guidewire or catheter is removed through the sheath. This step is associated with the majority of the bleeding complications that arise from percutaneous PVE, including hemoperitoneum, subcapsular hematoma, hepatic artery injury or pseudoaneurysm, and transient hemobilia (50, 51). If the puncture is made *via* an intercostal space, intercostal artery injury, hemothorax, or pneumothorax can also occur. Meticulous preprocedural evaluation of the peripheral portal branches to identify safe access, minimizing the number of needle passes and using a 21- or 22-gauge needle, can reduce the chance of complications during this step. The next step involves flush portography and portal pressure measurement. A 5-Fr flush angiographic catheter is introduced into the main portal vein over the guidewire. The catheter or guidewire that is inserted into the portal venous system must be manipulated as gently as possible so as not to cause any damage to the vessels that can result in thrombosis. This care is especially important when a contralateral approach is used. Flush portography is then performed to confirm the portal vein branching anatomy that was previously assessed with cross-sectional imaging and to determine the portal vein branches that must be embolized. At least two projections are often necessary to evaluate the portal vein branching anatomy fully; a craniocaudal or right anterior oblique projection is often added to an anterior–posterior projection for evaluation of the right posterior portal vein. Portal pressure is measured, especially in patients with cirrhosis, to confirm the absence of portal hypertension. Severe portal hypertension with a portosystemic pressure gradient greater than 12-mm Hg is a relative contraindication for major hepatic resection (52). If any portosystemic collaterals are identified (thus reflecting portal hypertension), the indication for PVE needs to be reconsidered. Once the portal vein branching anatomy and the absence of portal hypertension are confirmed, the portal branches within the liver segments to be resected are selected, and embolic materials are delivered until flow stasis is achieved. In the authors’ experience, where an ipsilateral approach is used, a 5-Fr reverse curved catheter (Sos Omni, AngioDynamics, Inc., Queensbury, NY, USA) is used to select the right portal vein branches. The most proximal sites of the portal vein branches where embolic material can be placed can vary, depending on the portal branching anatomy and the technique used for the anticipated hepatic resection. As discussed in the prior section, care should be taken to leave an approximately 1-cm portion of the proximal right portal vein free of embolic material, as the inflammatory reaction caused by embolic material can make the surgical division or suture ligation difficult. In cases of planned extended right hepatectomy, several researchers have advocated embolization of Sg 4 (Figure 9) to maximize the hypertrophy of the FLR (Sg 2 and 3) (53, 54). This step is usually performed prior to right PVE because embolic materials placed within the previously embolized right liver can potentially dislodge to the FLR during embolization of Sg 4 (55). Potential complications during this step include non-targeted embolization and left portal vein thrombosis. While delivering the embolic material to the targeted vessels, care must be taken not to cause backflow of the embolic material to the FLR especially when the antegrade flow within the targeted vessel becomes sluggish. Left portal vein thrombosis is an especially serious complication, as it can preclude future hepatic resection (56). If the thrombus is acute and extensive, chemical thrombolysis with or without mechanical thrombectomy should be considered. After embolization is complete, a repeat portogram is obtained to confirm adequate embolization of the portal vein branches within the liver segments to be resected. The portal pressure is again measured to check for the presence of portal hypertension, which might be uncovered following the procedure. Normally, the portal pressure increases by 3–5 mm Hg. Great care must be exercised not to dislodge the embolic material delivered during the portography. Because of the potential risk of dislodging embolic material, we normally do not exchange the working 5-Fr catheter for a flush angiographic catheter prior to portography. Finally, the catheter is gently removed from the portal venous system, with care taken to avoid propagation of any embolic materials to the FLR. Embolization of the access tract with Gelfoam^®^ or coils is optional but is commonly performed to prevent bleeding complications when an ipsilateral approach is used.

## Additional Strategies or Technical Modifications for PVE

**Figure 10:**
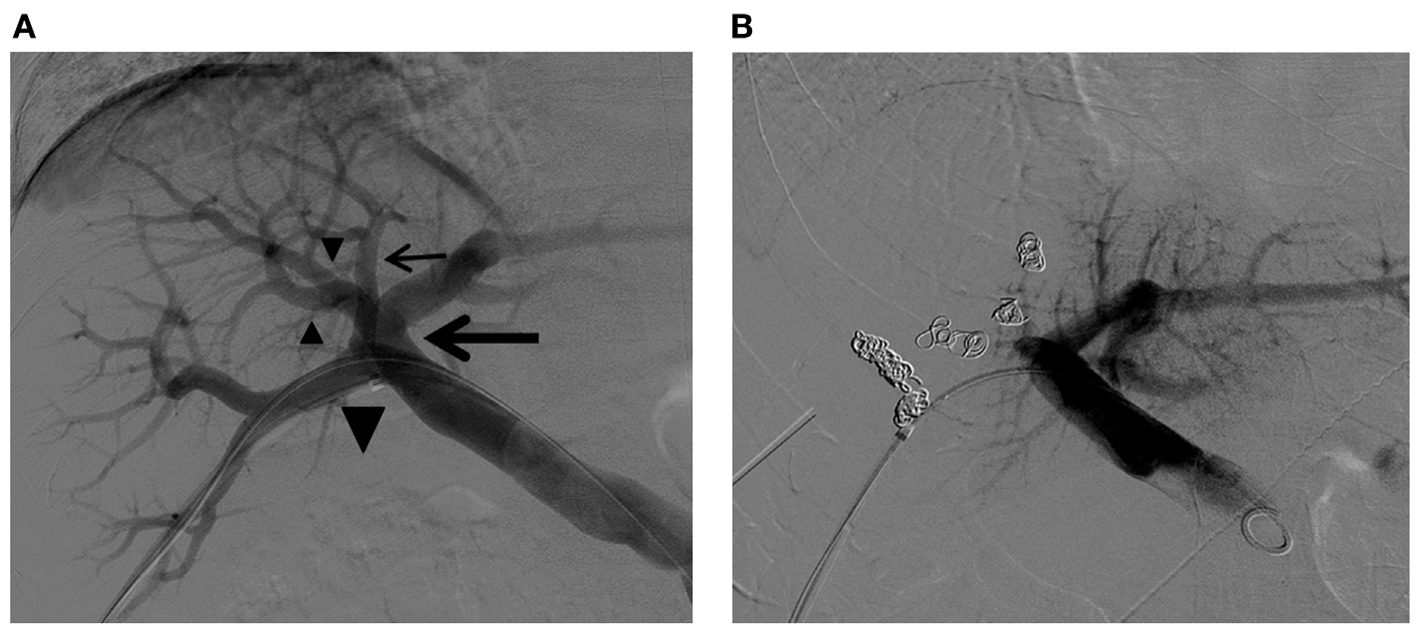
**Right PVE in a patient with complex right portal vein branching anatomy**. **(A)** Flush portogram shows the right posterior portal vein arising as the first branch of the main portal vein (large arrow). The Sg 7 branch arises from the proximal right posterior portal vein (small arrow). The common trunk (large arrowhead) of the left portal vein and the right anterior portal vein trifurcate into the left portal vein and two right anterior portal vein branches (small arrowheads). Based on this right portal vein branching anatomy, the four right portal vein branches are separately embolized with PVA particles and metallic coils. **(B)** Final portogram shows successful right PVE, with preserved blood flow to the left liver. Note the metallic coils placed in the proximal portion of each branch.

**Figure 11:**
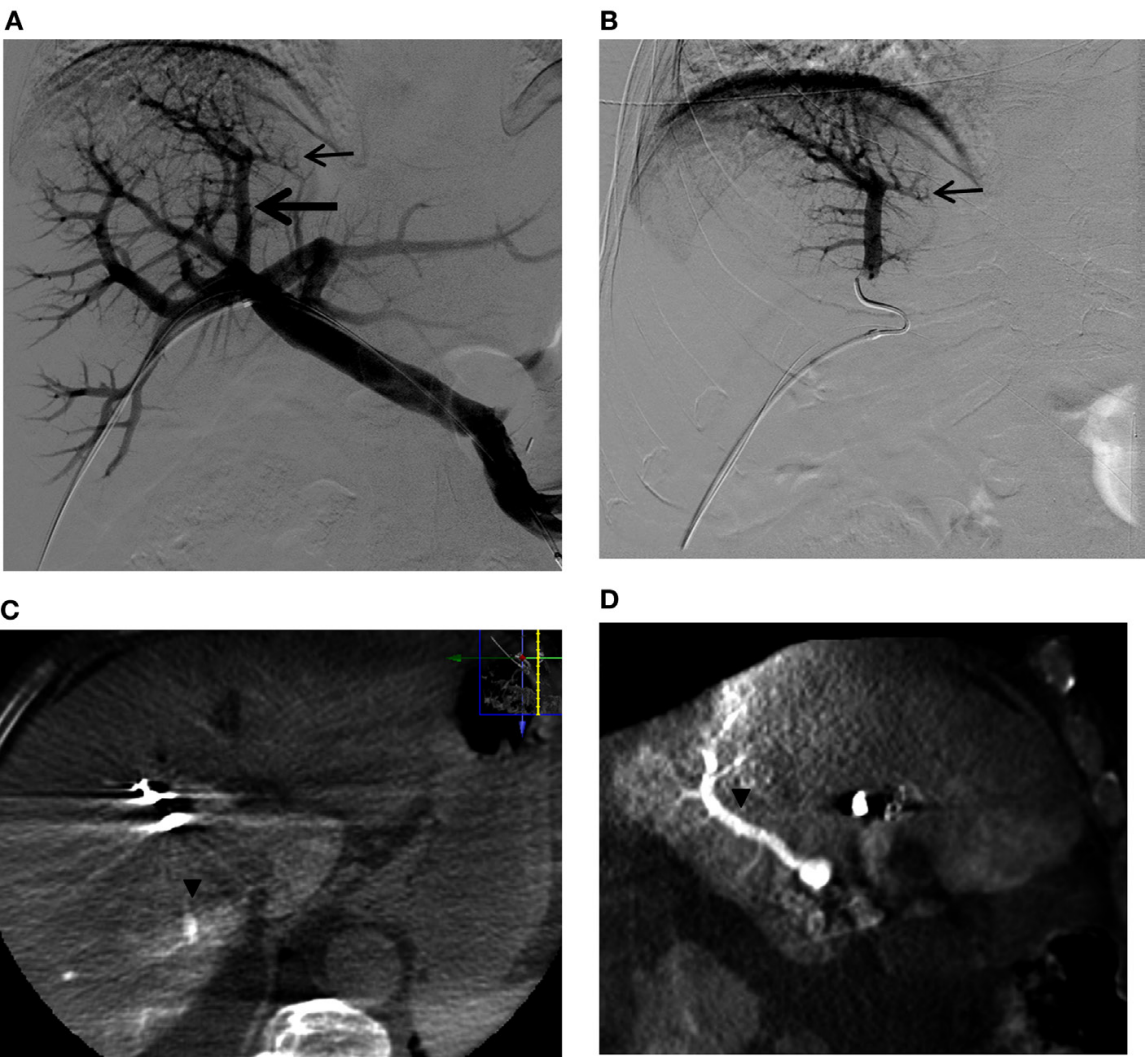
**A questionable subsegmental portal vein branch crossing the midplane of the liver in a patient referred for right PVE**. **(A)** Flush portogram shows a subsegmental branch directing to the left (small arrow) that is arising from the segmental branch directing to the dome of the liver (large arrow). **(B)** Selective right portogram clearly shows the subsegmental branch that possibly supplies segment 4 (small arrow). **(C,D)** Axial, **(A)** sagittal, and **(B)** reformatted C-arm cone-beam CT images during selective right portogram show the segmental branch supplying segment 7 (arrowheads). The subsegmental branch that was considered to be possibly supplying segment 4 was shown to be supplying segment 7. This segmental branch was then confidently embolized with PVA particles and coils. Note that artifact from the metallic coils placed in the right liver during the PVE procedure.

**Figure 12:**
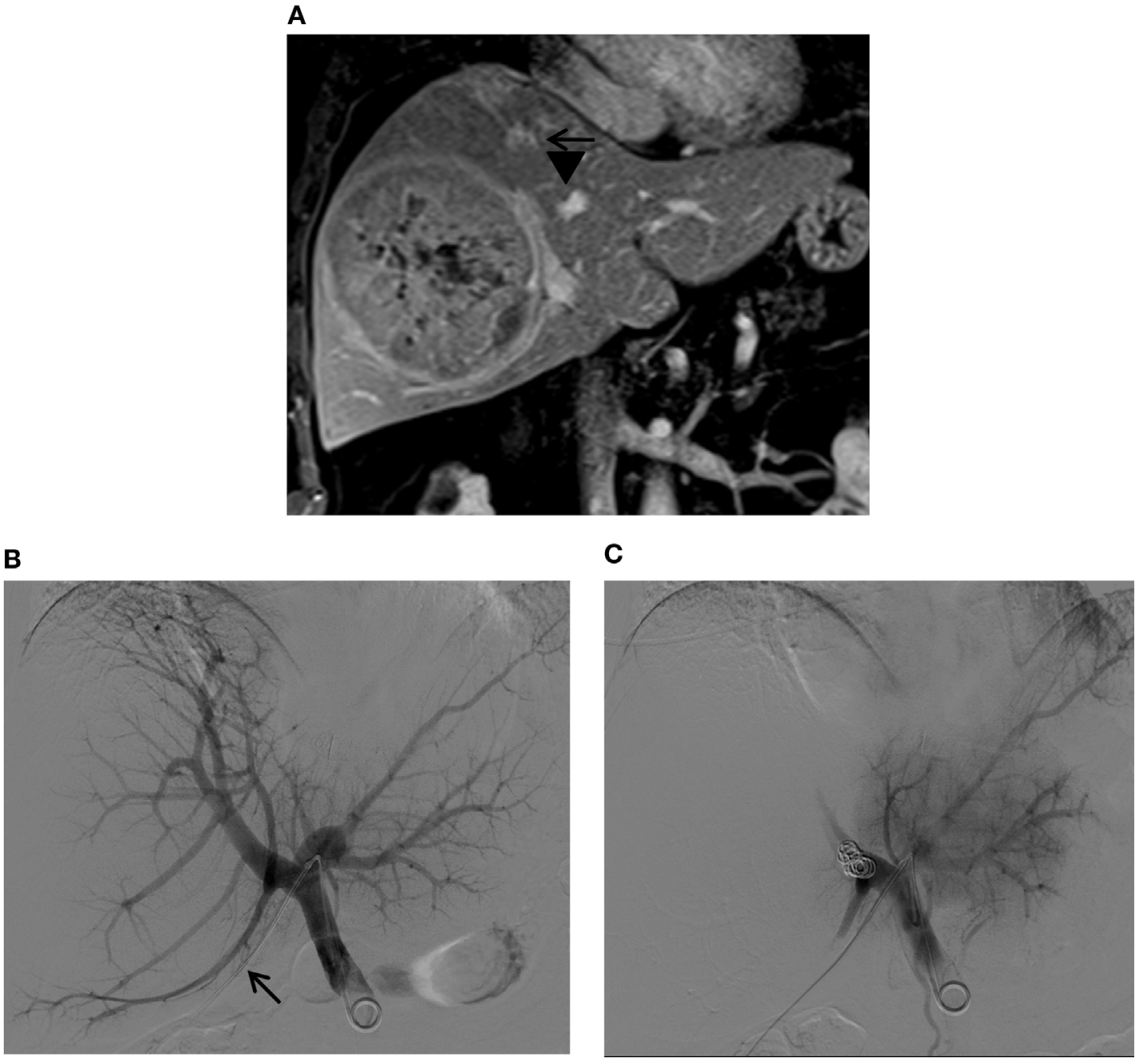
**Right PVE *via* a contralateral approach in a patient with a large hepatocellular carcinoma**. **(A)** Contrast-enhanced T1-weighted coronal MR images show a large tumor in the right liver. Marked distortions of the anterior (arrows) and posterior (arrowheads) branches of the right portal vein caused by the tumor are noted. The patient was referred for right PVE prior to a right hepatectomy. An ipsilateral approach was initially planned; however, no portal branches were identified for safe access within the right liver during the procedure. Access to the Sg4 portal vein branch was then obtained with a 22-gauge Chiba needle (not shown). **(B)** Flush portogram shows a 5-Fr pigtail catheter placed within the main portal vein through a 6-Fr sheath (arrow). **(C)** Final portogram shows successful occlusion of the right portal vein branches, with preserved blood flow to the left portal vein branches.

At times, additional strategies or technical modifications are required to accomplish preoperative PVE. Cases with complex portal vein branching anatomy can require selection and embolization of the individual variant portal vein branches (Figure 10). The portal branching anatomy must be meticulously assessed, and the portal access site should be carefully planned for easy cannulation of all of the variant branches that must be embolized. With some portal vein branches, especially when branching variations are present, it is difficult to assess which segment the branches are supplying, even with portograms from multiple projections. In such situations, C-arm cone-beam CT can easily depict the segment that the portal vein branch in question is supplying and can help to avoid non-targeted embolization or to allow for confident embolization of the selected branch (Figure 11) (57). Large tumors can pose a challenge for portal access and complete embolization of the portal branches within the diseased liver because of distortion of the portal vein branches by the tumor. Access through the tumor should be avoided, as tumor puncture can potentially result in hemoperitoneum or tumor seeding. An initial plan of an ipsilateral approach might need to be changed to a contralateral approach during the procedure because few portal vein branches are accessible within the diseased liver (Figure 12).

## Summary

Preoperative PVE is used to extend the indications for major hepatic resection and has become the standard of care for selected patients with hepatic malignancies in major hepatobiliary centers. To date, various techniques, using different embolic materials, have been employed without significant differences in the degree of liver hypertrophy. Both surgeons and interventional radiologists must be familiar with the segmental anatomy of the liver, have a good knowledge of portal vein anatomy and branching variations, and understand the techniques used to ligate the portal vein during planned hepatic resection because these variables can affect the PVE procedure and ultimately the surgical resection. Knowledge regarding the advantages and disadvantages of the approaches to the portal system and of the various embolic materials used for PVE is essential to best tailor the procedure and to avoid complications. Because PVE is used as an adjunct to planned hepatic resection, priority must always be placed on safety, without compromising the integrity of the FLR, and close collaboration between interventional radiologists and hepatobiliary surgeons is essential to achieve successful outcomes.

It is important to note that new innovative approaches are being developed that are variations on the standard PVE. For example, associating liver partition and portal vein ligation (ALPPS) is a technique involving hepatectomy in two stages. At the first stage, a hepatotomy through the anticipated resection plane is performed while preserving the exposed vessels and the portal vein to the portion of liver to be resected is surgically ligated. The FLR hypertrophies much faster than with standard PVE, in 1–2 weeks, after which the hepatic resection can be completed. A faster hypertrophy is seen in this procedure compared with standard PVE due to inflammatory signals from both the embolization and from the laparotomy with hepatic transection. Although there are higher hypertrophy rates with ALPSS compared to standard PVE, the rates of morbidity and mortality are significantly higher, limiting the routine applications of this technique currently (58, 59). Other approaches being investigated include radioembolization as a treatment for liver tumors. A recent review described how radioembolization of tumors can result in hypertrophy of the contralateral lobe, albeit slower than with PVE (60). This has implications for preoperative treatment of liver tumors in a fashion similar to PVE, although allowing for treatment of the tumor while causing hypertrophy of the contralateral lobe.

Despite these alternatives, the gold standard method presently remains PVE as the optimal oncosurgical strategy to allow for hypertrophy of the contralateral liver and make potentially unresectable lesions resectable by better selection and lower risk of postoperative liver insufficiency or failure. This strategy is ideal for high volume centers and should be performed in a multidisciplinary setting to allow for the highest chance of success for patients.

## Author Contributions

SO, KK, MS, BH, AS, BS, and DA – substantial contributions to the conception or design of the work; or the acquisition, analysis, or interpretation of data for the work; drafting the work or revising it critically for important intellectual content; final approval of the version to the published; and agreement to be accountable for all aspects of the work in ensuring that questions related to the accuracy or integrity of any part of the work are appropriately investigated and resolved.

## Conflict of Interest Statement

The authors declare that the research was conducted in the absence of any commercial or financial relationships that could be construed as a potential conflict of interest.
